# Comparative effects of nonsteroidal anti-inflammatory drugs at castration and tail-docking in neonatal piglets

**DOI:** 10.1371/journal.pone.0254409

**Published:** 2021-11-30

**Authors:** Emma Nixon, Alexandra R. Carlson, Patricia A. Routh, Liliana Hernandez, Glen W. Almond, Ronald E. Baynes, Kristen M. Messenger

**Affiliations:** 1 Department of Population Health and Pathobiology, College of Veterinary Medicine, North Carolina State University, Raleigh, NC, United States of America; 2 Department of Molecular Biomedical Sciences, College of Veterinary Medicine, North Carolina State University, Raleigh, NC, United States of America; University of Illinois, UNITED STATES

## Abstract

This study assessed the efficacy of meloxicam, flunixin, and ketoprofen in piglets undergoing routine castration and tail-docking. Six-day-old male piglets (8/group) received one of five randomized treatments: intramuscular saline (SAL PROC), meloxicam (MEL; 0.4 mg/kg), flunixin (FLU; 2.2 mg/kg), ketoprofen (KETO; 3.0 mg/kg) or sham (SAL SHAM; saline injection, no processing). Two hours post-dose, piglets were castrated and tail-docked. Plasma cortisol, interstitial fluid (ISF) prostaglandin E2 (PGE2) and activity levels via Actical® monitoring were used to estimate pain. SAL SHAM and FLU exhibited lower cortisol concentrations than SAL PROC at the time of processing (p = 0.003 and p = 0.049, respectively), and all NSAIDs exhibited lower PGE2 than SAL PROC at 3.69 hours (MEL p = 0.050; FLU p = 0.043 and KETO p = 0.031). While not statistically significant, PGE2 was higher in SAL PROC piglets vs. other treatment groups at most time points. There was also a high degree of variability between piglets, especially for SAL PROC. Activity levels were significantly decreased at multiple time points in SAL PROC and MEL piglets following processing. However, FLU and KETO piglets had increased activity levels closer to that of the SAL SHAM group, suggesting that these NSAIDs are more effective than MEL in providing analgesia. These results demonstrate that management strategies including administration of intramuscular flunixin or ketoprofen to reduce pain associated with processing will likely improve piglet health and welfare in the United States.

## Introduction

Piglets in the United States routinely undergo painful procedures, collectively known as processing, which can include castration, tail-docking, teeth clipping, ear notching/tagging, and injections. Castration (i.e., removal of the testicles or destruction of testicular formation) is commonly performed to prevent unwanted breeding, reduce aggression, and reduce boar taint (improving meat quality) [1] and tail docking (i.e., removal of a portion of the tail) is intended to reduce tail biting severity and total events [2]. To improve piglet welfare standards, Europe and Canada have implemented legislation requiring that piglets receive anesthetic or analgesic drugs in conjunction with processing procedures [3, 4]. Additionally, legislation is in place to prevent routine tail-docking in the EU unless there is evidence that tail biting has occurred [5].

In 2018, the reported number of pigs produced in the United States was approximately 133.5 million [6]. Assuming half of those pigs are male, an estimated 66.8 million piglets will likely undergo castration and tail docking each year. Castration and tail docking are often performed simultaneously; both cause tissue damage, and the acute consequences of these invasive procedures in terms of pain and stress are well described [7–19]. While not currently required in the United States, the provision of analgesia for piglets is a critical welfare issue, and many large retailers are requiring pain mitigation throughout their supply chains [20]. However, for legislation to be implemented to require pain management, drugs with proven efficacy and FDA approval are needed.

Data supporting the use of NSAIDs to manage pain is conflicting between studies [19, 21–25], which may be due to the doses or methodology used to assess efficacy. There is also only one other study that assessed the pharmacodynamics of multiple NSAIDs in direct comparison [26]. In that study, both ketoprofen and meloxicam were determined to be ineffective for castration-associated pain in piglets (Viscardi and Turner, 2018).

The objectives of the present study were to describe the pharmacodynamics of three different NSAIDs (meloxicam, flunixin, and ketoprofen) in neonatal piglets. This article only focuses on the comparative analgesic efficacy aspect of the study; the pharmacokinetics portion of the study is reported elsewhere [27]. Given that pain is a complex and multidimensional phenomenon, and there is conflicting data in the open literature on the efficacy of various NSAIDs at piglet processing, this study used a multimodal approach to assess the severity of pain and inflammation in piglets at castration and tail docking. This study achieved a direct comparison of three NSAIDs (meloxicam, ketoprofen, and flunixin), using objective measures of prostaglandin E2, cortisol, and activity levels, while also comparing to untreated controls (both processed and sham processed).

## Materials and methods

### Animals

This study was approved by the North Carolina State University Institutional Animal Care and Use Committee (protocol #17-088-A). A total of 46 Yorkshire/Landrace cross male piglets (weighing 3.80–3.21 kg and from 14 different litters) were individually housed in cages arranged so they were able to see each other. Lighting consisted of 12 hours of light and 12 hours of dark and ambient room temperature was maintained at 26–30 degrees Celsius. Heat lamps were hung above the piglets on one end of the individual cages. Piglets were fed non-medicated swine milk replacer (Milk Specialties Global, Eden Prairie, MN, USA) and offered fresh water every 4 hours from 7 am to 12 am. Piglets were weighed daily on a calibrated scale (i.e., 48 and 24 hours pre-dose and 0, 24, and 48 hours post-dose). The piglets were humanely euthanized at the end of the study. First, the piglets were sedated via intramuscular injection of 50:50 ketamine (100 mg/mL) and xylazine (100 mg/mL) equivalent to a final dose of 2.2 mg/kg ketamine and 2.2 mg/kg xylazine. After sedation, Euthasol® was administered through the jugular vein catheter at a dose equivalent to 85.9 mg/kg pentobarbital sodium and 11 mg/kg phenytoin sodium. Death was confirmed by thoracic auscultation (lack of both heart and respiratory sounds), as well as lack of a corneal reflex.

### Catheter and interstitial fluid probe placement

At 4 days-of-age (±1 day), a catheter (22 Ga, 10 cm small animal long-term venous catheterization kit, MILA International, Inc., Florence, KY, USA) was placed aseptically into the jugular vein under sevoflurane anesthesia. At the time of catheter placement, an ultrafiltration probe (RUF-3-12 Reinforced In Vivo Ultrafiltration Sampling Probes, BASi systems, W. LaFayette, IN, USA) was placed subcutaneously along the epaxial muscles. Details for each procedure were previously reported [27]. Finally, activity monitors (Actical, Philips Respironics, Bend, OR, USA) were also secured to the back of the piglets’ neck with Ioban™ (3M, St. Paul, MN, USA). Piglets were then allowed to recover for 36–48 hours before the start of the study. During the recovery period, catheter patency was maintained by removing the heparin lock, flushing the catheter with saline, and replacing the heparin lock every 12 hours.

### Drug administration and processing

In the present study, “processing” was defined as castration and tail-docking only. At 6 days-of-age (±1 day) piglets were randomized using a random number generator and allocated to one of five treatment groups; Saline with sham processing (SAL SHAM; n = 9), saline with processing (SAL PROC; n = 8), meloxicam and processing (MEL; n = 8), flunixin and processing (FLU; n = 7) and ketoprofen and processing (KETO; n = 8). Forty out of 46 piglets completed the study. Six piglets were removed from the data analysis due to illnesses unrelated to the study (severe diarrhea, malaise, abdominal cyst), and their data were not included in the results. Two piglets used in a pilot study were not included due to differences in housing and handling.

All treatments were administered intramuscularly as follows; One hundred microliters of saline (both SAL SHAM and SAL PROC), 0.4 mg/kg meloxicam (Meloxicam solution for injection 5 mg/mL, Putney, Inc., Portland, ME, USA), 2.2 mg/kg flunixin (Banamine-S®, Merck Animal Health, Summit, NJ, USA) or 3.0 mg/kg ketoprofen (Ketofen®, Zoetis, Inc., Kalamazoo, MI, USA). These doses were chosen based on existing labels for piglets at castration (meloxicam and ketoprofen) in Europe and an existing label for other indications in pigs (flunixin) in the United States.

Two hours after drug administration, one researcher restrained the piglets to expose the anogenital region and a second person (with extensive experience in piglet processing procedures) performed the castration and tail-docking. Incisions were made with a scalpel on each side of the scrotum, the testicles were pulled from the surrounding tissue and the scalpel was used to cut the testicles free. Side-cutter pliers were used to dock the tail per industry standard in the United States. Then, the castration site and tail were sprayed with Betadine® surgical scrub (Emerson Healthcare LLC., Wayne, PA, USA) to disinfect the wounds. SAL SHAM piglets were handled for approximately the same length of time as other pigs, and handled similarly, but not castrated or tail docked.

### Sample collection

Blood samples (1 mL) were collected via the jugular catheter and transferred into lithium heparin tubes (BD Vacutainer^TM^, Becton, Dickinson and Company, Franklin Lakes, NJ, USA) at the following time points: 0 (baseline), 0.25, 0.5, 1, 1.5, 2, 4, 6, 8, 12, 24, 36 and 48 hours post-dose. Plasma was collected by centrifugation of the blood at 3500 x g and used for the analysis of plasma cortisol concentrations. Interstitial fluid samples were collected at 0 (baseline), 2, 4, 6, 8, 12, 24, 36, and 48 hours post-dose and used to quantify prostaglandin E2, a biomarker of inflammation. A lag time for ISF collection was calculated based on the length of the probe and the rate of fluid collection from each probe. The ultrafiltration probes are 42.5 cm in length and hold 70 μL of fluid, and the probes were cut to custom lengths for each animal which was accounted for in the lag time calculation. Both plasma and ISF were frozen at -80°C until analysis.

### Plasma cortisol analysis

Plasma cortisol samples were analyzed using a commercial radioimmunoassay (RIA) kit (ImmuChem™ Cortisol Coated Tube RIA Kit, MP Biomedicals, LLC., CA, USA). The samples were assayed in triplicate and analyzed on a Packard Cobra gamma counter. Calibration curves were within the range of 1.0–25.0 μg/dL and all R^2^ values were >0.9970. The inter-day assay variability was 2.95 ±1.15% and the intra-day assay variability was 7.88 ±8.36%.

### Interstitial fluid prostaglandin E2 analysis

The concentration of interstitial fluid prostaglandin E2 (PGE2) was determined using a commercially available enzyme-linked immunosorbent assay (ELISA) kit (Cayman Chemical, Co., Ann Arbor, MI, USA). R^2^ for all calibration curves were >0.96 and within the range of 7.81–1000 pg/mL. The inter-day assay variability was 10.8% and the intra-day assay variability was 3.1%. All samples were analyzed in duplicate.

### Activity monitoring

Actical monitors were placed on the back of the neck at the time of catheter and interstitial fluid probe placement. Actical monitors are 17 g omnidirectional accelerometers which collect data each minute on spontaneous activity. The activity monitors were active from the morning of catheter and probe placement, but only the data from 24 hours prior to drug administration, until 48 hours after drug administration were analyzed. The 24 hours immediately before dose were used as the baseline. The Actical monitors provide an output of activity counts when the data are downloaded.

### Statistical analysis

All statistical analyses were performed in GraphPad Prism (version 8.4.3, GraphPad Software, Inc., San Diego, CA, USA). All data were tested for normality by the Shapiro-Wilks test. PGE2 data were not normally distributed but found to be log-normally distributed, and therefore were log-transformed before statistical testing. All data except activity were analyzed using a mixed model procedure, including treatment, time, and treatment x time interaction. Time was a repeated measure with piglet as the experimental unit. A post hoc Tukey’s test was conducted for significant outcomes.

### Activity count statistical analysis

For statistical analysis, activity was split into three 24-hour periods; baseline, 0–24 h post-dose, and 24–48 h post-dose. Similarly to a previous study analyzing activity data [28], total activity counts for 6-hour periods were calculated and described as quarters (Q1, 08:00–13:59; Q2, 14:00–19:59; Q3, 20:00–01:59; and Q4, 02:00–07:59). The values for each quarter post-dose were compared to the baseline values within each treatment group. Data were analyzed using a mixed model procedure, with multiple comparisons and Tukey’s test for significant outcomes. Time was a repeated measure with piglet as the experimental unit. The treatment groups were also compared to the control (SAL SHAM) at each time point. This was also analyzed using a mixed model procedure but this time with Dunnett’s post hoc test.

In addition to the above, a functional linear modeling (FLM) approach was also used. This type of analysis is designed for actigraphy time-series data, allowing statistical characterization of activity patterns, while avoiding summary statistics that risk masking differences between groups and changes in patterns over time [29, 30]. This analysis was performed using R software [31] and the package “actigraphy” [32], which applies a non-parametric permutation F test.

Significance was calculated by counting the proportion of permutation F values that are larger than the F statistics for the observed pairing. In this case, a point-wise test (bspline method with 500 permutations) was used to generate a curve representing the proportion of all permutation F values at each point in the time series [29, 30, 33].

## Results

### Plasma cortisol

**Fig 1:**
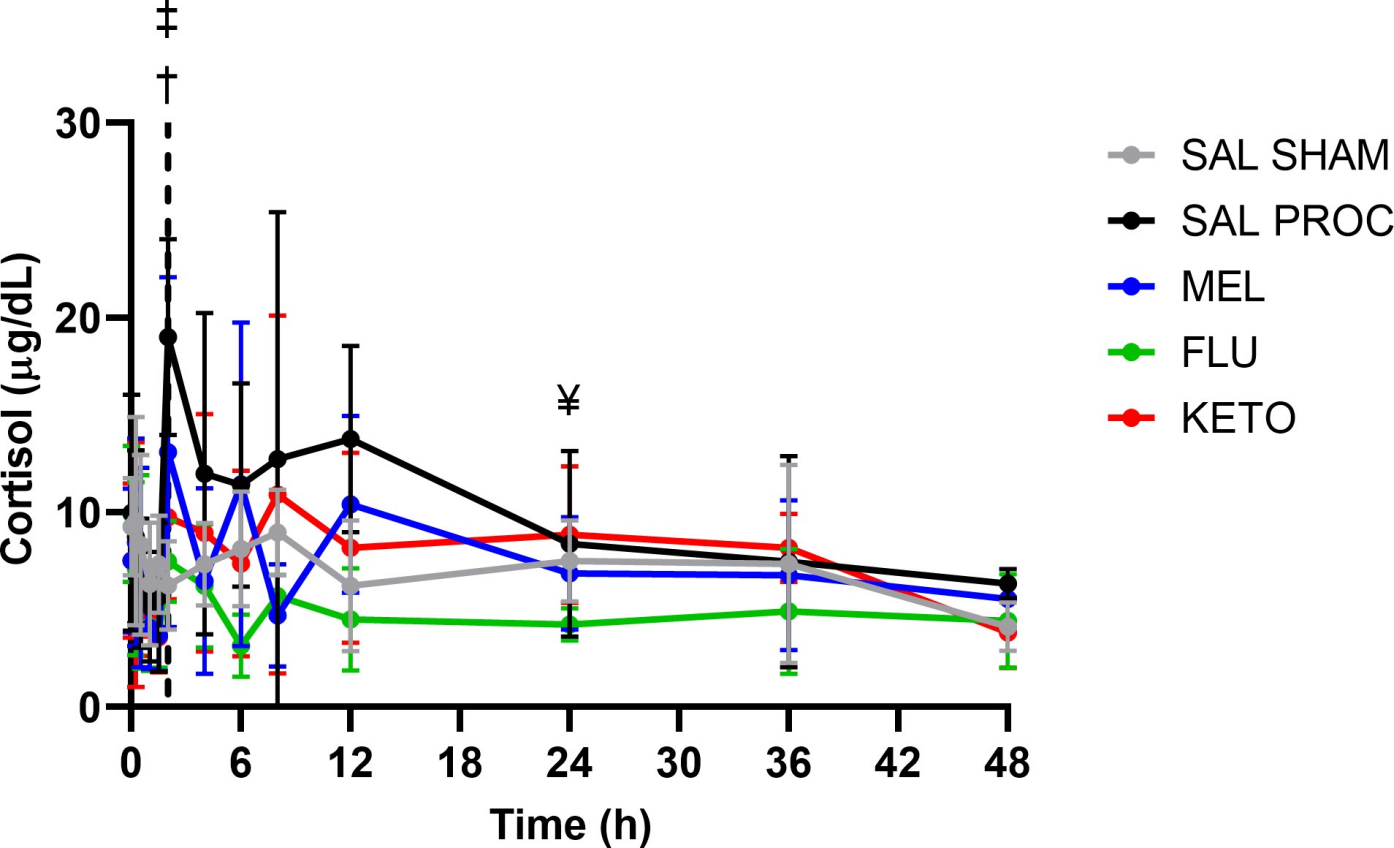
Total plasma cortisol concentration over time following intramuscular administration of either 0.1 mL saline, 0.4 mg/kg meloxicam, 2.2 mg/kg flunixin or 3.0 mg/kg ketoprofen in 6-day-old piglets. All piglets were processed (castrated and tail-docked) at 2 h as shown by the vertical dotted line, except for the SAL SHAM group. Data are represented as mean ± standard deviation. † indicates significant difference between SAL PROC and SAL SHAM; ‡ indicates significant difference between SAL PROC and FLU; ¥ indicates significant difference between FLU and KETO.

There was an effect of time (p = 0.001) but not treatment (p = 0.315) or the interaction between time and treatment (p = 0.209) on plasma cortisol concentrations. However, for the pairwise comparisons, both the SAL SHAM and FLU groups exhibited significantly lower plasma cortisol levels at 2 h than the SAL PROC group (p = 0.003 and p = 0.049, respectively). FLU piglets also had significantly lower cortisol than KETO piglets at 24 h (p = 0.022). The plasma cortisol results for each group are presented in Fig 1.

### ISF prostaglandin E2

**Fig 2:**
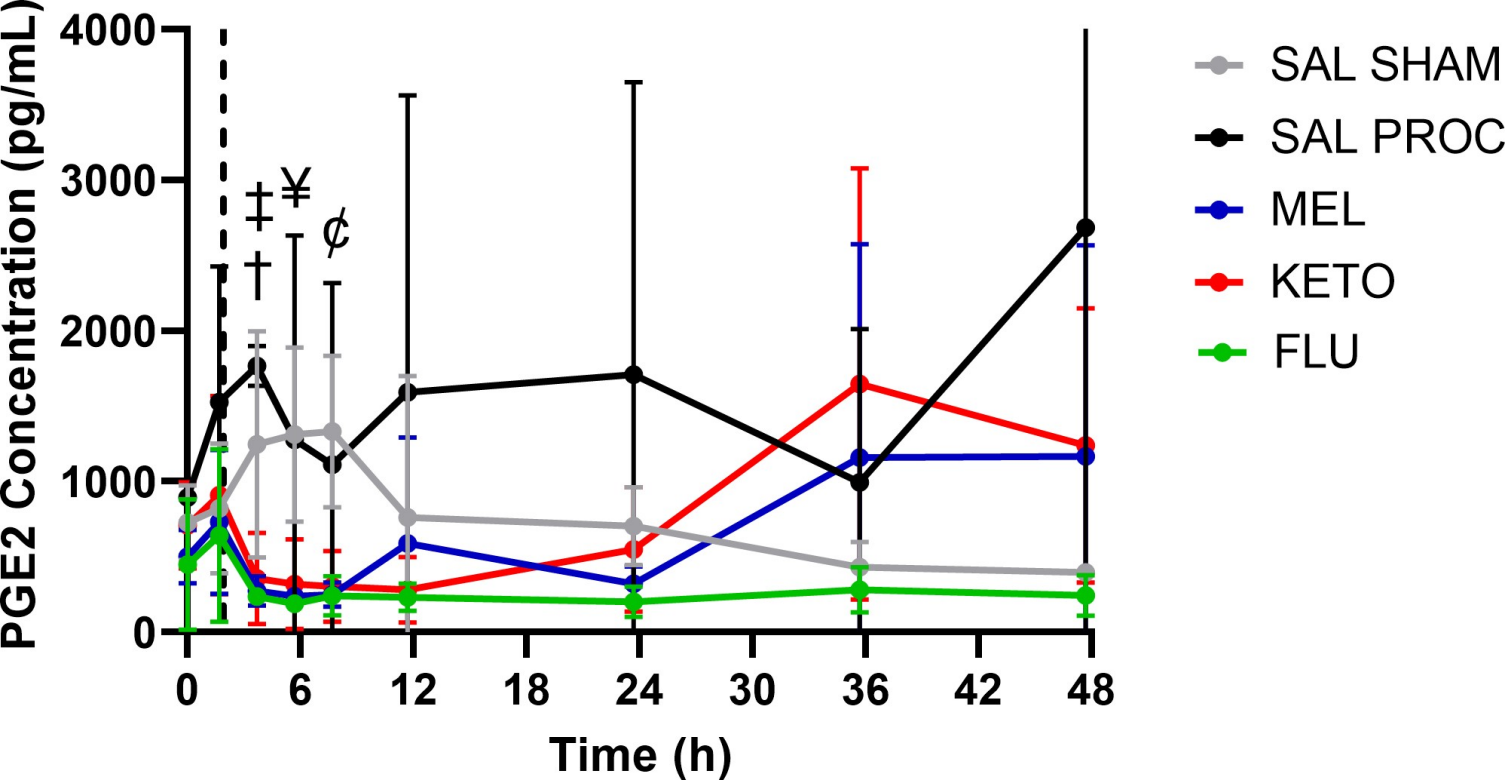
Total ISF PGE2 concentration over time (accounting for ISF probe lag time) following intramuscular administration of either 0.1 mL saline, 0.4 mg/kg meloxicam, 2.2 mg/kg flunixin or 3.0 mg/kg ketoprofen in 6-day-old piglets. All piglets were processed (castrated and tail-docked) at 2 h as shown by the vertical dotted line, except for the SAL SHAM group. Data are represented as mean ± standard deviation. † indicates significant difference between SAL PROC and all NSAID groups; ‡ indicates significant difference between SAL SHAM and MEL/FLU; ¥ indicates significant difference between SAL SHAM and MEL/FLU; Ȼ indicates significant difference between SAL SHAM and all NSAID groups.

The calculated lag time for the interstitial fluid collection was 0.31 hours. That is, it takes 0.31 hours for the sample to travel along the tubing, and this is accounted for in the results. The effect of treatment was significant (p = 0.048), but the effect of time or the interaction between treatment and time was not significant (p>0.05). All NSAID treatments exhibited statistically lower PGE2 at 3.69 hours post-dose than the SAL PROC group (MEL p = 0.050; FLU p = 0.043 and KETO p = 0.031), and the MEL and FLU groups also had lower PGE2 than the SAL SHAM group at 3.69 (MEL p = 0.050 and FLU p = 0.043), 5.69 (MEL p = 0.020 and FLU p = 0.008) and 7.69 hours (MEL p = 0.012 and FLU p = 0.020). KETO also had lower PGE2 than the SAL SHAM group at 7.69 hours (p = 0.043). While not statistically significant, SAL PROC PGE2 concentrations were higher than that of other treatment groups at most time points. There was also high variability in PGE2 concentrations between piglets, especially for the SAL PROC group. The ISF PGE2 results for each group are presented in Fig 2.

### Activity

**Fig 3:**
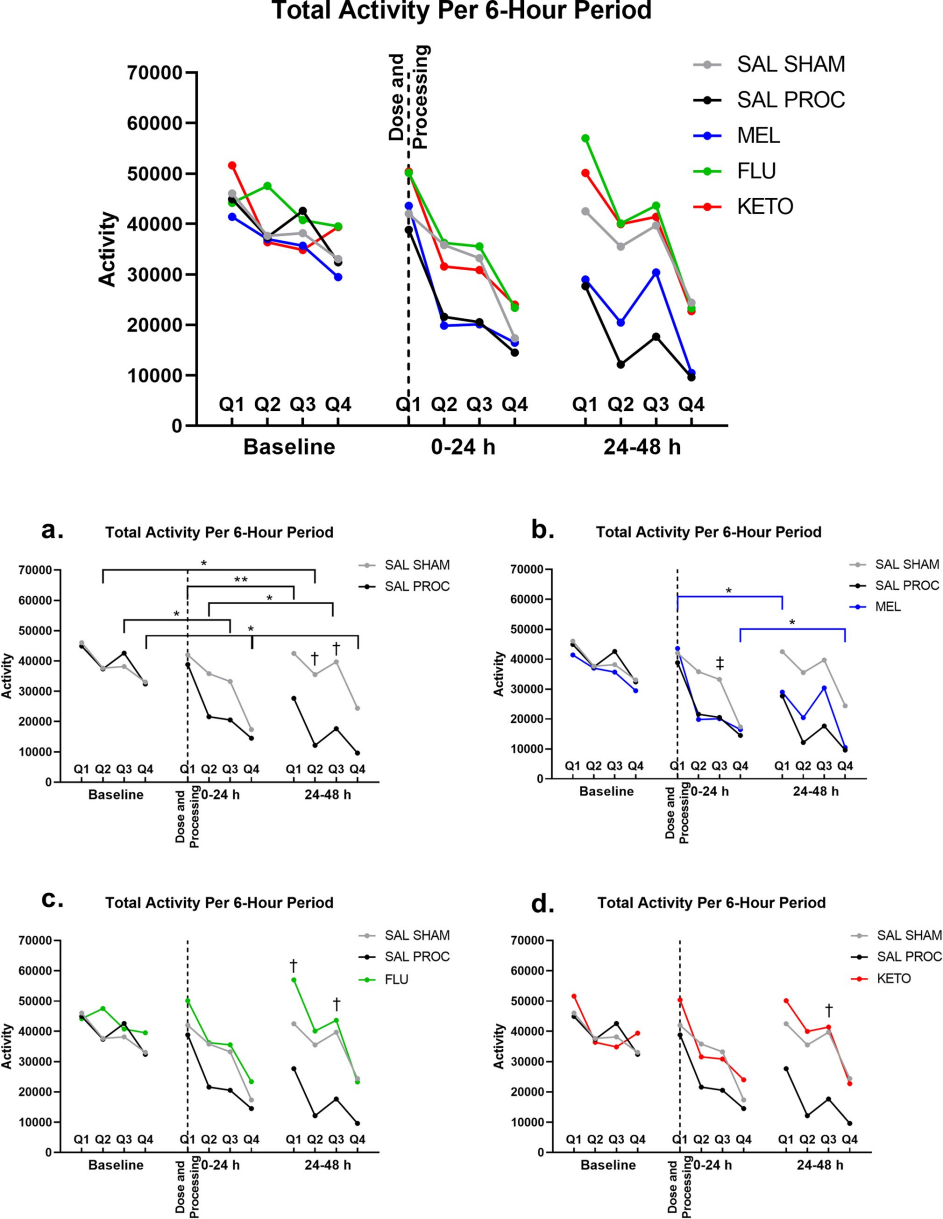
a: Shows the total activity levels for each quarter (Q, 6-hour period) Q1, 8:00–13:59; Q2, 14:00–19:59; Q3, 20:00–01:59; Q4, 02:00–07:59. b: The same data as Fig 3A but divided by treatment. Total activity levels for each quarter (Q, 6-hour period) Q1, 8:00–13:59; Q2, 14:00–19:59; Q3, 20:00–01:59; Q4, 02:00–07:59. * indicates significant difference (p<0.05) between the indicated time periods for SAL PROC (graph a) and MEL (graph b). ‡ indicates significant difference between MEL and SAL SHAM (p<0.05). † indicates significant difference between SAL PROC and SAL SHAM (graph a) or NSAID-treated group (graphs b-d).

For activity levels, the mixed model showed that treatment and time were statistically significant (p = 0.035 and p<0.001, respectively; Fig 3A and 3B). SAL PROC and MEL piglets at 0-24h Q3 and Q4 had significantly lower activity compared to baseline at Q3 (SAL PROC, p = 0.032; MEL, p = 0.048) and Q4 (SAL PROC, p = 0.023; MEL, p = 0.023). However, FLU and KETO piglets were significantly more active when compared to SAL PROC at 24–48 h Q3 (p = 0.012 and p = 0.024, respectively). FLU piglets were also significantly more active than SAL PROC piglets at 24–48 h Q1 (p = 0.027). Finally, the FLU group was significantly more active at 24–48 h compared to 0–24 h at Q3 (p = 0.014).

**Fig 4:**
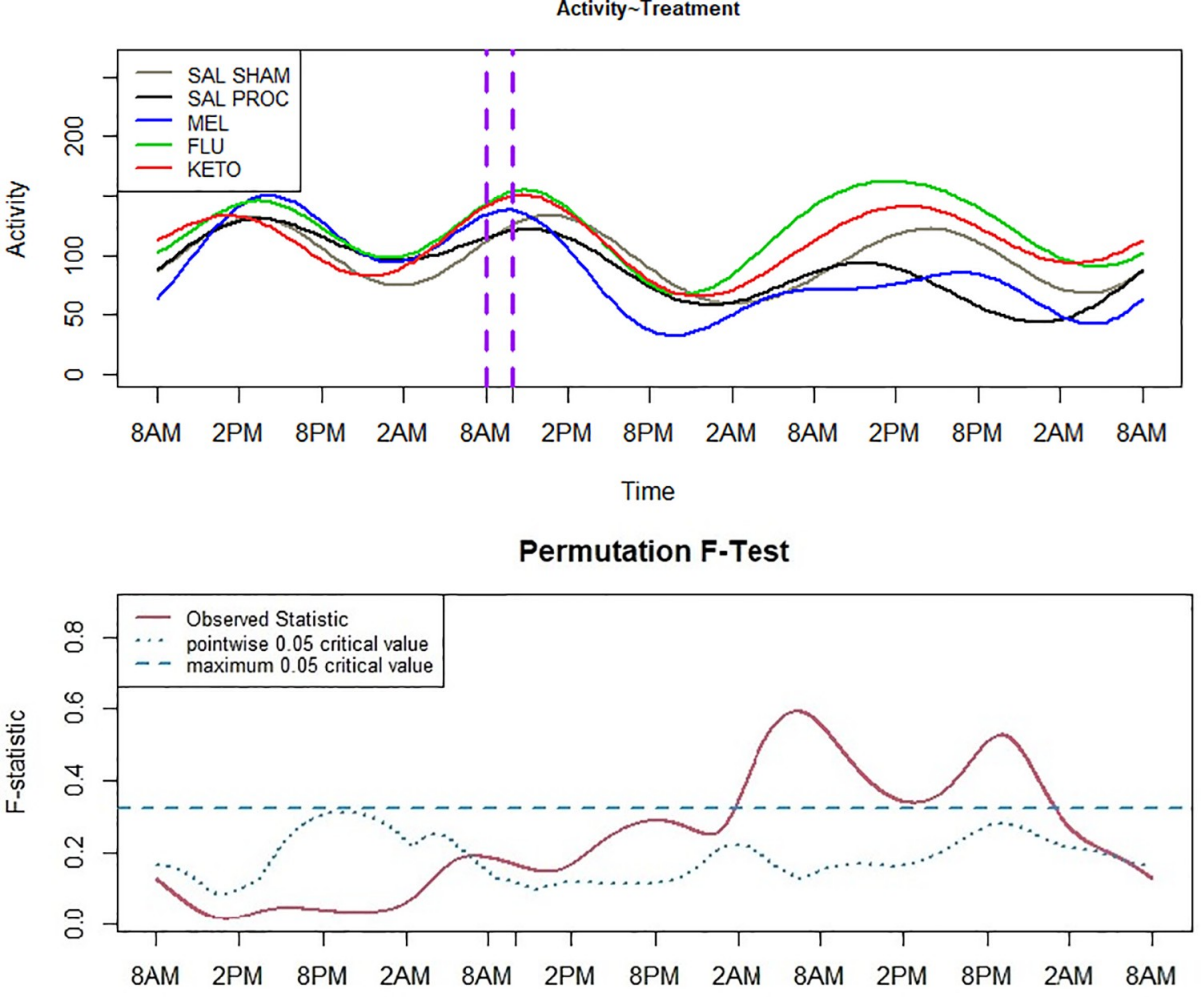
Functional linear modeling (FLM) of activity data following intramuscular administration of either 0.1 mL saline, 0.4 mg/kg meloxicam, 2.2 mg/kg flunixin or 3.0 mg/kg ketoprofen in 6-day-old piglets. All injections were given at the time represented by the first vertical dashed line, and piglets were processed (castrated and tail-docked) 2 hours later, as shown by the second vertical dashed line, except for the SAL SHAM group. The bottom panel shows the point-wise critical value (dotted line), the proportion of all permutation F values at each time point at the significance level of 0.05. When the observed F-statistic (solid red line) are above the dotted line, the groups are considered significantly different.

The FLM demonstrated changes in 24-hour activity patterns after processing, particularly for the SAL PROC and MEL treatment groups between approximately 18–42 hours after dosing (16–40 hours post-processing; Fig 4). FLU and KETO appeared to be effective in maintaining sleep-wake patterns closer to that of baseline, similar to the SAL SHAM piglets, and overall activity was reduced for the SAL PROC and MEL piglets.

### Bodyweight

**Fig 5:**
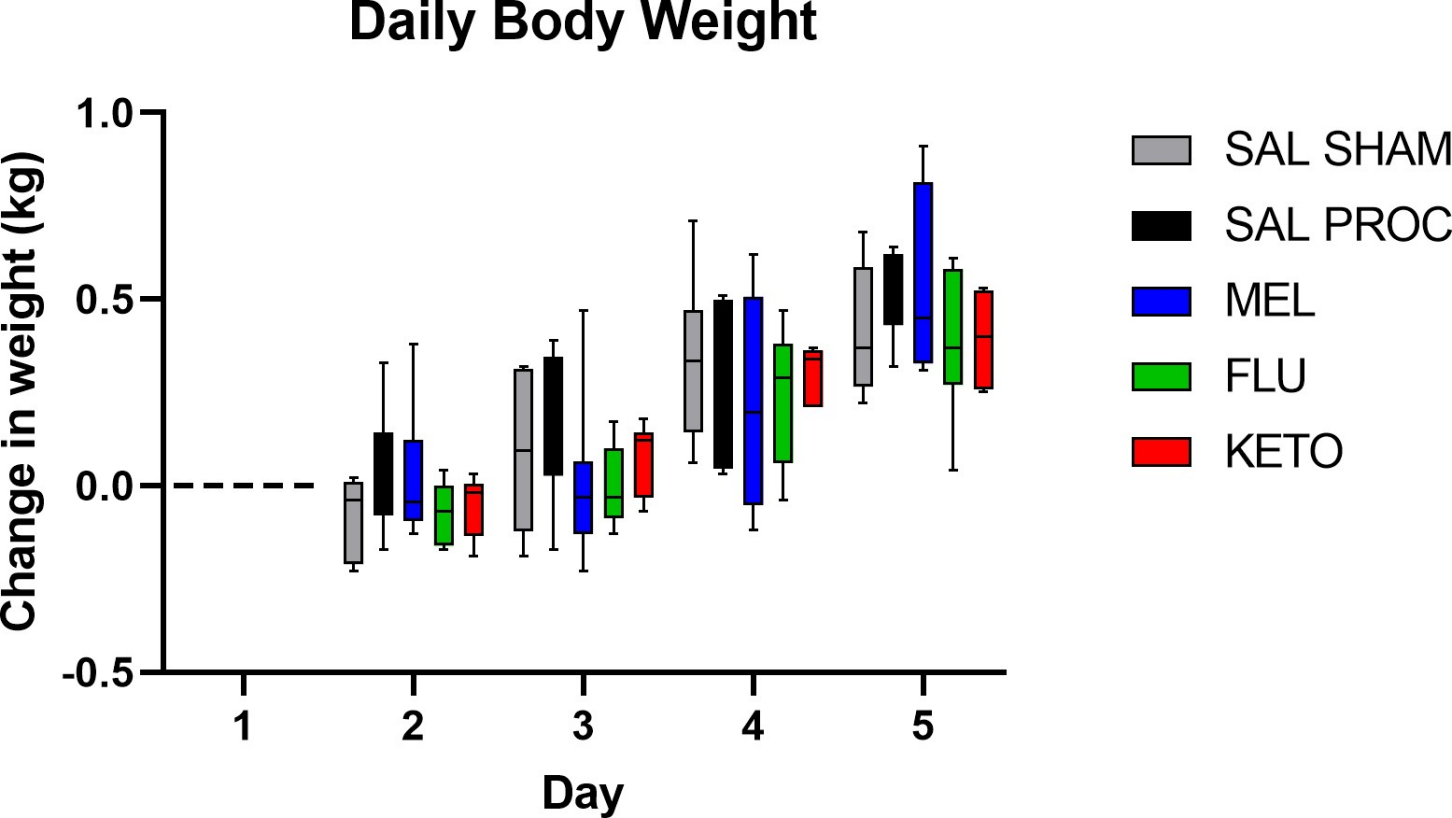
Daily change in bodyweight from baseline over the course of the study, where day 0 is when catheters and ISF probes were placed, and day 3 is the day of processing.

Daily change in body weight was not significantly different between treatment groups (Fig 5). All piglets gained weight over the course of the study regardless of treatment.

## Discussion

NSAIDs are commonly administered analgesics for treating pain at piglet processing, however, there are conflicting data supporting the use of NSAIDs across studies [19, 21–25]. The present study is unique in that it provides a direct comparison between three different NSAIDs simultaneously, as only one other study has directly compared the pharmacodynamics of two NSAIDs at piglet processing [26]. The results of this study demonstrated that flunixin may be the most effective NSAID in alleviating processing-associated pain in piglets, followed by ketoprofen. Similarly to previous reports [34], meloxicam showed minimal efficacy at the EU/Canada label dose. The sham-processed piglets showed negligible effects of handling or intramuscular injection (saline) on the measured outcomes. There was a small increase in PGE2 following the injection, which returned to baseline in under 12 hours. The activity was also slightly reduced overnight after saline administration and sham processing, but again, quickly returned to baseline by 24 hours.

While the utility of measuring cortisol as a proxy for pain is controversial, cortisol was included in this study as it has been commonly assessed in previous piglet pain studies. As expected, SAL PROC piglets had significantly higher plasma cortisol concentrations immediately after processing compared to SAL SHAM piglets. Overall, flunixin achieved the greatest reduction in plasma cortisol throughout the duration of the study, although this was only statistically significant at 24 hours after drug administration (22 hours after processing). All NSAIDs also decreased PGE2 compared to SAL PROC soon after processing, but flunixin maintained this inhibition beyond 24 hours post-dose. This longer duration of action is consistent with the fact that flunixin has a longer half-life than meloxicam or ketoprofen in pigs [27].

Accelerometry has been used in other species to measure activity levels in relation to painful conditions [30, 35, 36], and in sows to detect lameness [37–39]. However, to the best of the author’s knowledge, the present study is the first to use accelerometry in assessing piglet pain. Activity levels were significantly decreased in SAL PROC and MEL piglets following processing, while FLU and KETO treated pigs had increased activity levels, closer to that of the SAL SHAM group suggesting that these NSAIDs are more effective than meloxicam in the provision of analgesia. In addition, following FLM, the 24-hour activity patterns appear to be altered following processing, with significant differences between approximately 18–42 hours after dosing (16–40 hours post-processing). The results suggest that these changes may be mitigated by treating with flunixin or ketoprofen.

Finally, there were no significant differences in weight gain in the first 48 hours after dosing. While not significantly different, flunixin and ketoprofen-treated piglets appeared to gain slightly less weight than meloxicam-treated piglets. This could indicate a link between increased activity and decreased weight gain. Weight gain may not be a helpful measure for determining pain as painful piglets are more likely to have reduced activity, therefore reduced calorie expenditure and possibly increased weight gain. There is conflicting evidence to support whether analgesia/anesthesia can improve weight gain following castration, and very few efficacy studies measured the long-term weight gain of piglets. Of those that did, different analgesics/anesthetics were administered, so it is difficult to draw any meaningful conclusions [40–42]. Future studies would need to monitor weight gain and activity for a longer time to determine any longer-term effects, as the meloxicam-treated piglets in this study had not returned to baseline activity levels by 48 hours (the end of the study).

### Limitations

This study has some limitations worth consideration. Piglets were removed from their litters/sows and individually housed. While this is not reflective of farm management, it was necessary to maintain patency of IV catheters/ISF probes and to prevent damage to the accelerometer. Removal from the sow, individual housing and use of milk replacer are all possible sources of additional stress, and this must be considered before attempting to extrapolate the results of this study to on-farm conditions. All piglets were treated, handled, and housed identically; thus, it is unlikely that this limitation affected the primary outcomes (i.e., the comparison of meloxicam, flunixin and ketoprofen *within* this study), however, on-farm efficacy studies would need to be performed to validate these results. Additionally, NSAIDs were administered 2 hours before processing. In a production setting, to administer a drug and then return 2 hours later may not be practical. This timing was chosen purposefully to ensure NSAID concentrations would reach maximum levels in the tissues- one of the main sites of anti-inflammatory and analgesic actions of these drugs- by the time of processing [27]. Finally, it is difficult to differentiate between stress, pain, and inflammation, and none of the measures used in this study directly measure ‘pain’, which can also be a subjective experience. However, in combination, these objective measures can provide some insight into the analgesic effects of these NSAIDs.

## Conclusion

In summary, flunixin (2.2 mg/kg) or ketoprofen (3.0 mg/kg) administered intramuscularly 2 hours before processing, were shown to be the most efficacious treatments in this study within the limitation of the three objective parameters that were assessed for analgesic effects. Additionally, meloxicam appears to be inferior as an NSAID analgesic to prevent pain associated with piglet processing (at a dose of 0.4 mg/kg), despite its current use in the EU and Canada. Similar findings have been reported by other investigators [26]. This study was the first to investigate changes in activity associated with processing using a wearable accelerometer in piglets. These results demonstrate that management strategies including administration of intramuscular flunixin or ketoprofen to reduce pain associated with processing will likely improve piglet health and welfare in the United States. Future investigation will involve PK/PD modeling to correlate plasma and tissue (interstitial fluid) concentrations with efficacy, based on the promising parameters in this paper, with the goal of establishing a standard in drug concentrations and physiological/behavioral outcomes to which other analgesics can be compared.

## Supporting information

S1 Data (XLSX)
